# Optimization of Spiral-Wound Microfiltration Process Parameters for the Production of Micellar Casein Concentrate

**DOI:** 10.3390/membranes11090656

**Published:** 2021-08-26

**Authors:** Chenchaiah Marella, Venkateswarlu Sunkesula, Ahmed R. A. Hammam, Anil Kommineni, Lloyd E. Metzger

**Affiliations:** 1 Midwest Dairy Foods Research Center, Dairy and Food Science Department, South Dakota State University, Brookings, SD 57007, USA; vsunkesula@idahomilk.us (V.S.); ahmed.hammam@sdstate.edu (A.R.A.H.); anil.kommineni@danone.com (A.K.); Lloyd.Metzger@sdstate.edu (L.E.M.); 2 Idaho Milk Products, Jerome, ID 83338, USA

**Keywords:** polymeric spiral-wound membrane, microfiltration, transmembrane pressure, diafiltration, micellar casein concentrate

## Abstract

A systematic selection of different transmembrane pressures (TMP) and levels of diafiltration (DF) was studied to optimize these critical process parameters during the manufacturing of micellar casein concentrate (MCC) using spiral-wound polymeric membrane filtration. Three TMPs (34.5, 62.1, and 103.4 kPa) and four DF levels (0, 70, 100, and 150%) were applied in the study. The effect of the TMP and DF level on flux rates, serum protein (SP) removal, the casein-to-total-protein ratio, the casein-to-true-protein ratio, and the rejection of casein and SP were evaluated. At all transmembrane pressures, the overall flux increased with increases in the DF level. The impact of DF on the overall flux was more pronounced at lower pressures than at higher pressures. With controlled DF, the instantaneous flux was maintained within 80% of the initial flux for the entire process run. The combination of 34.5 kPa and a DF level of 150% resulted in 81.45% SP removal, and a casein-to-true-protein ratio of 0.96. SP removal data from the lab-scale experiments were fitted into a mathematical model using DF levels and the square of TMPs as factors. The model developed in this study could predict SP removal within 90–95% of actual SP removal achieved from the pilot plant experiments.

## 1. Introduction

Membrane separation technology was introduced to dairy processing in the early 1970s as an alternative to some thermal and non-thermal processes [1]. Commonly used membrane processes in the dairy industry are microfiltration (MF), ultrafiltration (UF), nanofiltration (NF), and reverse osmosis (RO). The MF process uses porous membranes with a porosity of 0.1 to 2 µm [2] and is extensively used in the defatting of whey stream in the production of whey protein isolates [3]. The interest in using MF in micellar casein concentrate (MCC) production has increased recently [4,5,6,7,8]. The MCC is produced from the MF of skim milk by permeating most of the serum protein (SP) and non-protein nitrogen components, thereby increasing the ratio of casein to total protein (CN/TKN) and casein to true protein (CN/TP). The retentate obtained from this process is a concentrated colloidal suspension [9] containing casein in micellar form, lactose, minerals, and some serum proteins. MCC has been utilized in some applications, such as cheese making [7,10,11], acid curd [12], process cheese [7,13,14,15,16], imitation mozzarella cheese [17], and Greek-style yogurt [7,18]. MCC has other promising applications, such as nutritional meal replacements, whipped toppings, and coffee whiteners. The permeate obtained from this process is another ideal starting material for manufacturing native serum protein concentrates, native α-lactalbumin, and β-lactoglobulin-enriched protein ingredients.

Most of the research on the MF of skim milk for MCC production has used ceramic MF membranes [6,8,19,20]. Ceramic membrane systems are capital intensive, and membrane replacements are expensive [7,21,22]. Compared to these systems, membrane separation systems using polymeric membranes have a smaller footprint, are inexpensive, and are familiar to most of the US dairy processors. In recent years, the interest in assessing the suitability and efficiency of polymeric MF membranes for MCC production has been increased [5,7,21,23,24,25,26,27]. It has been shown that using ceramic membranes, over 95% of serum protein could be removed in a three-stage process in which diafiltration (DF) to a level of 200% (on the basis of feed volume) was used. DF is a process in which water is added to the retentate during MF and further concentration is carried out. This step is intended to improve the serum protein (SP) removal and to control the membrane polarization phenomenon [7,28]. Studies conducted on the use of polymeric membranes for the production of MCC have shown that approximately 40% of SP is removed from skim milk at the first stage of MF [5,21] and the cumulative SP removal increases to around 60% in the second stage, when 200% of DF water is added to the retentate of the first stage [5]. In this process, skim milk is concentrated to three times (3X) volume reduction (VR) and water equal to the original milk volume is added to the retentate. Further filtration is done to a VR of 3X, and this process is repeated twice, totaling 200% DF. While this shows the positive impact of DF on process efficiency, it is essential to retain the membrane permeation characteristics over extended process runs to maximize the SP removal. During the MF of skim milk, concentrations of materials retained by the membrane continue to increase with process time. Higher concentrations of these materials in the bulk of the fluid lead to a more pronounced concentration-polarization of the membrane or fouling, thereby negatively impacting the serum protein permeation through the membrane [7,28]. This effect could be seen when only 40% of SP could be removed during MF without adding DF water, and this percentage could be increased to 70% with DF water. When DF water is added to the retentate, the bulk concentration of material goes down, thereby improving the membrane permeation characteristics. A controlled DF addition should help minimize the concentration-polarization effect and maximize SP removal in this process.

Besides DF, transmembrane pressure (TMP) is also an important operating variable in the MF process. TMP is the driving force in the MF process and is the most pressure-sensitive of all the membrane processes. Using higher pressure leads to higher initial flux rates but causes increased concentration polarization or fouling, leading to a gradual decrease in flux rates and membrane permeation characteristics [28]. The formation of a polarization layer and subsequent compaction of this layer and the membrane at higher pressures lead to the pressure-independent operation of MF. Therefore, to sustain a reasonable flux rate over a longer process run and to maintain membrane permeation characteristics, the selection of suitable TMP is very important.

Thus, the objective of the present study is to evaluate the spiral-wound (SW) MF process for maximizing SP removal by optimizing the operating pressure and the DF level.

## 2. Materials and Methods

### 2.1. Feed

Fresh skim milk was collected from the South Dakota State University dairy plant. The skim milk was pasteurized at 63 °C for 30 min. For different replications, skim milk collected on different days was utilized. 

### 2.2. Membranes

Polyvinylidene fluoride (PVDF) membranes in a flat sheet configuration (Parker Process Advanced Filtration Division, Oxnard, CA, USA) were used in the lab-scale MF experiments. The membrane sheets were cut to the required size of 14.0 cm× 15.2 cm for use in an OPTISEP membrane unit. Before using, all the flat sheet membranes were kept wet with 1% sodium metabisulfite solution and stored at 5 °C. For pilot plant experiments, two 0.5 µm PVDF membranes in SW configuration connected in parallel were used (element model FH 3030-OS03S). Each element was 97 mm in diameter and 762 mm in length. The elements had 1.1 mm spacers with a membrane area of 4.3 m^2^ per element. 

### 2.3. Proximate Analysis

Samples of feed, permeate, and retentate were analyzed for total solids (TS) using direct forced-air oven drying [29] (AOAC, 2000; method 990.20). Total protein nitrogen (TKN), noncasein nitrogen (NCN) and non-protein nitrogen (NPN) were determined by the block digester method using a micro-Kjeldahl apparatus. The true protein (TP) was calculated as the difference between TKN and NPN. Casein (CN) was calculated as the difference between TKN and NCN. The SP content was calculated as the difference between NCN and NPN. A multiplication factor of 6.38 was used to convert nitrogen to protein. 

### 2.4. SP Removal

The SP removal was calculated using micro-Kjeldahl data and the quantities of skim milk used and permeate collected from the process. The SP removal (%) was calculated by dividing the SP content of permeate (g) by the SP content (g) of the original skim milk and multiplying by 100. 

### 2.5. Overall Flux

Overall flux (O flux) was calculated as permeate flow rate per unit filtration area per unit time and is expressed as liters per meter square per hour (LMH).

$$\mathrm{Overall}\;\mathrm{flux},\;\mathrm{LMH}=\frac{\mathrm{Permeate}\;\mathrm{flow}\;[\mathrm{mL}]\;\times 60}{\mathrm{Collection}\;\mathrm{time}\;[\min]\;\times \mathrm{Area}\;\mathrm{of}\;\mathrm{membrane}\;[m^{2}]\;\times 1000}$$

In the present study, O flux was averaged over the entire process time. The feed volume and VR used in the study resulted in a process time of about 2 to 6 h.

### 2.6. Rejection Coefficients for SP and CN

Classical rejection coefficients are generally calculated as the ratio of concentration (C) of any component in permeate (p) and retentate (r). With processes using DF water, the concentration of any component in the permeate goes down due to the dilution effect. This gives a negative rejection coefficient for that component. To overcome this situation, in the present study rejection coefficients were calculated differently. The pore size of the membrane used in the experiments was 0.5 µ. Theoretically, all the components present in the milk should pass through the membrane resulting in 0 rejections [28]. This corresponds to an equal concentration of the component both in permeate and retentate. In the present study, rejection of a component is expressed as:

$$\mathrm{Rej}=1-\frac{Cp}{Cf}$$

where Cp is the concentration of any component in the permeate while Cf is the concentration of the component in the feed after adjusting for the level of DF water. For example, if skim milk had 0.54% serum protein and if an experimental run used 100% diafiltration, the adjusted concentration of serum protein in feed (Cf) would be 0.54/2 = 0.27%. 

### 2.7. Operating Variables

Transmembrane pressure (TMP) is the driving force in the MF of skim milk. As discussed in the preceding sections, the magnitude of TMP affects the nature of the pseudo filtration layer (concentration-polarization layer) formed on the membrane surface. A higher TMP leads to more compaction of this layer, which impacts the membrane permeation characteristics. To study the effect of TMP, experiments were conducted at three levels of TMP (34.5, 62.1, and 103.4 kPa). The TMP was read using an Ashcroft industrial Duralife pressure gauge with a measuring range of 0–413.7 kPa. 

TMP is the difference between the average of inlet and outlet pressures minus the permeate pressure.

$$\mathrm{TMP}=\frac{P_{\mathrm{in}}+P_{\mathrm{out}}}{2}-P_{p}$$

Diafiltration (DF) is often used in the MF process to maximize the protein recovery in the defatting of whey streams and in the MF of skim milk to maximize the SP removal, so that the CN to TP ratio in MCC can be increased. In the present study, three levels of DF (70, 100, and 150%, based on the feed volume) were used. A control run without any DF was also used to compare the effectiveness of DF in improving the efficiency of the MF process.

### 2.8. Experimental Procedure

**Figure 1:**
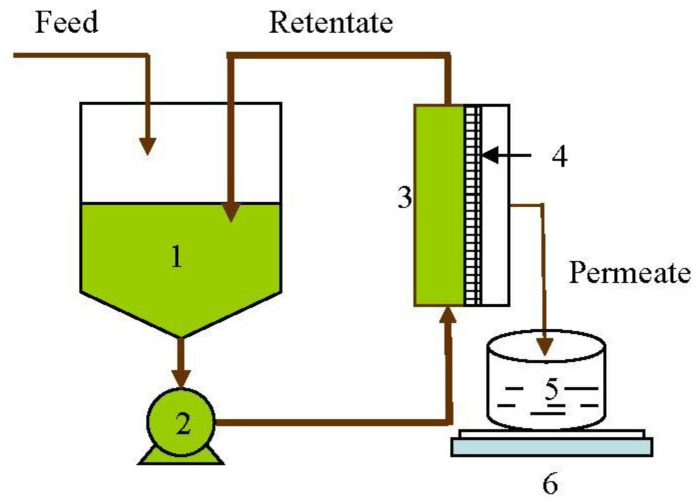
Schematic diagram of the lab-scale membrane separation process. 1—Feed tank; 2—Pump; 3—Flat sheet unit; 4—Membrane; 5—Permeate collection tank; 6—Weighing balance.

Lab-scale studies: MF experiments were conducted using a lab-scale plate-and-frame unit (OPTISEP 400-unit part # 20-000-1000) procured from NCSRT, NC. The unit used flat sheet membranes of 14 cm× 15.2 cm size with a filtration area of 0.02 m^2^. The gasket provided a channel height of 0.5 mm for the feed channel. The TMP was measured using the Ashcroft industrial Duralife pressure gauge (Ascroft, Stratford, CT06614, measuring range of 0–413.7 kPa) fitted to the end plate. The pressure was varied by controlling the back-pressure valve provided on the retentate line at the outlet of the unit. A variable-speed peristaltic pump (Masterflex peristaltic pump, Cat. # EW-77521-40, Cole Parmer, IL, USA) coupled to a standard L/S pump head (Cat # C-07024-21) was used to supply the feed to the membrane unit. The feed flow rate to the membrane unit was maintained at 1.7 L/min. Flat sheet membranes were cut into 14 cm× 15.2 cm pieces and were assembled in the filtration unit as per the instruction manual supplied with the filtration unit. Before each run, the membrane was flushed with 6 L of deionized water to flush out the storage solvent. Each batch was begun with a feed volume of 800 mL, and separation was conducted on continuous concentration mode as shown in Figure 1, to a final retentate volume of 200 mL, giving a VR of 4. All the experiments were conducted at a temperature of 24 °C and a pH of 6.6.

### 2.9. Statistical Analysis

All the data were analyzed with one-way ANOVA to test for significant differences among the treatments, with a type I error rate (α) of 0.05, using MINITAB^®^ 19 (Minitab, LLC, Chicago, IL, USA).

## 3. Results and Discussion

### 3.1. Flux

**Figure 2:**
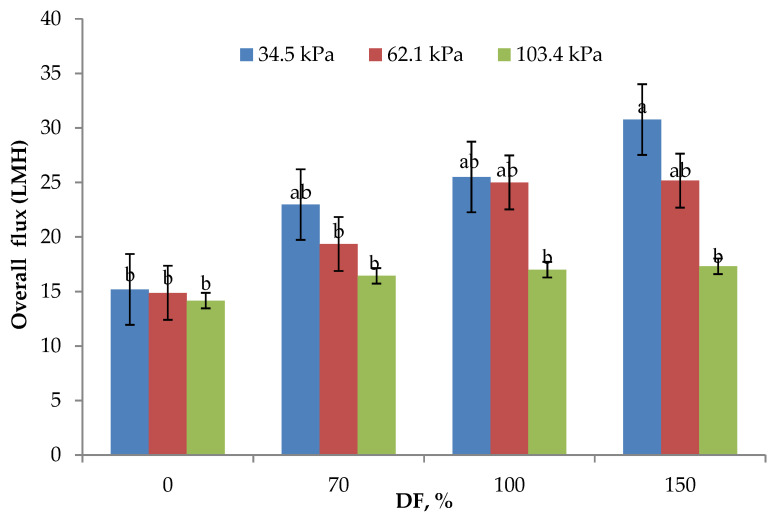
Overall flux means (*n* = 3) obtained from the lab-scale studies. All experiments were conducted at 24 °C and 800 mL feed was concentrated to a final volume of 200 mL resulting in a fourfold volume reduction (VR). DF is diafiltration water added, measured as a percentage of original feed volume. ^a,b^ Means not sharing the same letter are significantly different (*p* < 0.05).

The data on O flux obtained at all levels of DF and for all the pressures used in the study are presented in Figure 2. Both TMP and DF used in the study had a significant effect on O flux. This observation is similar to the results reported by Salunke and others [3]. The highest O flux of 23.61 LMH was obtained at the lowest TMP used in the study. As the TMP increased, O flux decreased. However, the O flux obtained at 34.5 and 62.1 kPa TMP (23.61 and 21.1 LMH, respectively) was statistically not different (*p* > 0.05). It has been reported that the use of 50 kPa TMP while manufacturing casein concentrate utilizing polymeric membranes did not affect the flux significantly [24], which is similar to the results obtained in this study. As shown in Figure 2, addition of DF water had a positive impact on the O flux. O flux increased by 50% from 0 to 70% DF at 34.5 kPa TMP, while the increase at the same TMP was about 100% from 0 to 150% DF. For the control run (0% DF), there was no significant difference observed in O flux for all the pressures used in the study and was about 14.75 ± 0.30 LMH. This shows that, it is essential to select appropriate pressure and DF level for optimum performance of MF. From the data presented, it is also clear that at higher operating pressures, DF becomes less effective in influencing the process flux rates. The lowest flux rates obtained in control runs at all the three pressures may be because, as higher concentrations of rejected solids build up in the retentate, the polarization of membrane becomes more pronounced and dictates the permeation characteristics of the membrane, making the process operate in a pressure-independent region [3,7]. DF showed a considerable influence on the process flux rates, especially at the lower pressures used in the study. It was reported that the DF water increased the flux in stage three as compared to stage one of MF [5].

**Figure 3:**
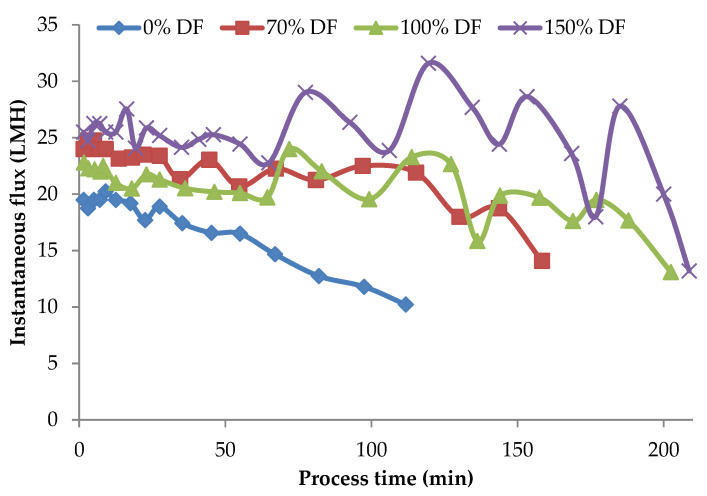
Instantaneous flux obtained from the lab-scale studies at 34.5 kPa pressure. Instantaneous fluxes are arrived at by measuring the permeate at various time intervals over the entire process run and averaging for that time interval. All the experiments were conducted at 24 °C and 800 mL feed was concentrated to a final volume of 200 mL resulting in a volume reduction of 4 (VR). DF is the percentage of diafiltration water based on the original feed volume.

Instantaneous flux rates obtained during the lab-scale run for 34.5 kPa pressure at all DF levels are presented in Figure 3. From the data presented in Figure 3, in the case of the control run the flux values continued to drop over the entire process run-duration of about 2 h. Zulewska et al. reported that the flux of the SW system decreased with increasing processing time [21]. This was also similar to Beckman’s study, which found that flux is decreased during three-stage MF processing [5]. For the other runs, DF water was added at a VR of 1.23, 1.45, 1.68, 1.88, 1.88, and 2.67. After the final addition at a VR of 2.67, the product was concentrated until a VR of 4 was reached. The valleys and peaks in the flux graphs are due to addition of DF water at different intervals. For control run, the initial flux was about 19.5 LMH; by the end of 3 VR (a concentration factor of three), the flux was only 60% of the initial flux. For all the DF runs, the flux at 3 VR was about 78–80% of the initial flux. The drop in flux from its initial value after about 90–95% of the process run-time was roughly 5–10%. The addition of DF in small quantities over several intervals of the process run helped sustain the process flux rate for a longer time. Additionally, the level of DF had a strong effect on the flux rates, resulting in higher flux rates with higher DF level.

**Figure 4:**
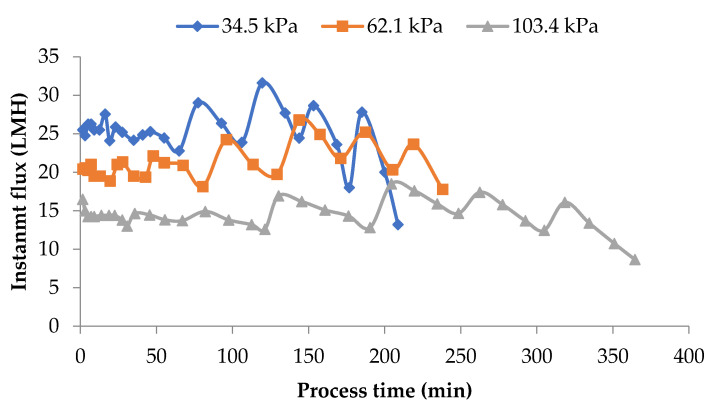
Instantaneous flux obtained from the lab-scale studies at the three pressures used. Diafiltration was 150% of feed volume and was added at 6 intervals. Instantaneous fluxes are arrived at by measuring the permeate at various time intervals over the entire process run and averaging for that time interval. All the experiments were conducted at 24 °C and 800 mL feed was concentrated to a final volume of 200 mL resulting in a volume reduction of 4 (VR). DF is the percentage of diafiltration based on the original feed volume.

Data presented in Figure 4 shows the impact of operating pressure on flux rates. From the data presented, the operating pressure showed a strong impact on the flux. The flux at all time intervals was about 50% higher for process runs that used 34.5 kPa pressure when compared to the run that used 103.4 kPa pressure. As highlighted in the earlier sections, MF is a pressure-sensitive process. Higher pressures promote a more pronounced polarization of the membrane. Furthermore, there is a compaction of this layer and the membrane leading to reduced flux. In the 103.4 kPa case, these phenomena may be overshadowing the advantage of DF, thereby maintaining the difference in flux among the three pressures used in the study.

### 3.2. SP Removal

**Figure 5:**
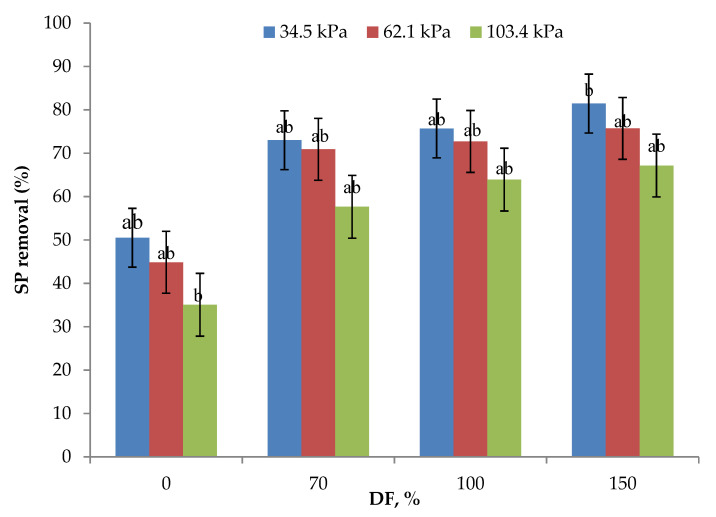
Data on serum protein (SP) removal obtained from the lab-scale studies at three pressures and for all levels of diafiltration used. Diafiltration (DF) water was on the basis of feed volume and was added at 6 intervals during the process. All the experiments were conducted at 24 °C and 800 mL feed was concentrated to a final volume of 200 mL resulting in a volume reduction of 4 (VR). ^a,b^ Means not sharing the same letter are significantly different (*p* < 0.05).

Serum protein removal data from the experiments are presented in Figure 5. Without DF, SP removal ranged from 35 to 50%, the highest being for 34.5 kPa and the lowest being for 106.4 kPa pressure. The statistical analysis of data showed that the SP removal obtained at 34.5 and 62.1 kPa TMP at 0, 70, and 100% DF were not statistically different (*p* > 0.05). At all the levels of DF, SP removal decreased with increases in the operating pressure. As discussed in the preceding sections, concentration polarization and compaction of the polarized layer, as well as the membrane, may determine the membrane permeation characteristics [23,30] and mask the beneficial effect of DF at higher operating pressures. The use of 70% DF at 34.5 kPa gave the same SP removal rate as that of 100% DF at 62.1 kPa pressure. Additionally, 100% DF at 34.5 kPa pressure gave a similar SP removal rate as compared to 150% DF at 62.1 kPa pressure. The highest SP removal of 81.45% was obtained with the use of 34.5 kPa pressure and 150% DF. These results highlight the importance of the selection of an appropriate operating pressure and level of DF for maximizing SP removal. A similar trend was found by Hurt and Barbano when studying the processing conditions of MF to remove SP [28]. They reported that increasing the DF and CF 5× led to an elevation of SP removal from 88.66 to 99.47%.

The SP removal data obtained from the lab-scale runs were fitted into a mathematical model and expressed as a function of the DF level and the square of TMP.

SP R% = 58.705 − 0.0015 × TMP2 + 0.18 × DF

where TMP is in kPa and DF is % of water added based on feed volume. Using the model, it is possible to predict SP removal at any selected operating condition.

### 3.3. Rejection of SP and CN

The data on CN/TKN, CN/TP ratios, rejection of CN (rej CN) and SP (rej SP) are presented in Table 1. In skim milk, the CN/TKN and CN/TP ratios were 0.77–0.79 and 0.8–0.83, respectively. The CN/TKN ratio ranged from 0.87 to 0.96; the highest ratio of 0.96 was obtained with 34.5 kPa pressure and 150% DF. The CN/TP ratio ranged from 0.89 to 0.96, the highest ratio of 0.96 was obtained with the use of 34.5 kPa TMP and 150% DF. For this combination, the highest SP removal was obtained. From the data, rej SP increased with operating pressure and decreased with the level of DF. The lowest rej SP of 0.1 was obtained with 34.5 kPa TMP and 150% DF combination. For all the runs, rej CN ranged from 0.97 to 1.0 by the membrane. The casein content of permeates ranged from 0.01 to 0.04%. The high rejection of SP could have resulted from fouling on the membrane, which has more effect with a lower DF level [28]. Beckman and others found that high TMP led to a higher SP rejection [5,23], which we noticed at all DF levels.

### 3.4. Pilot-Scale Experiments

**Figure 6:**
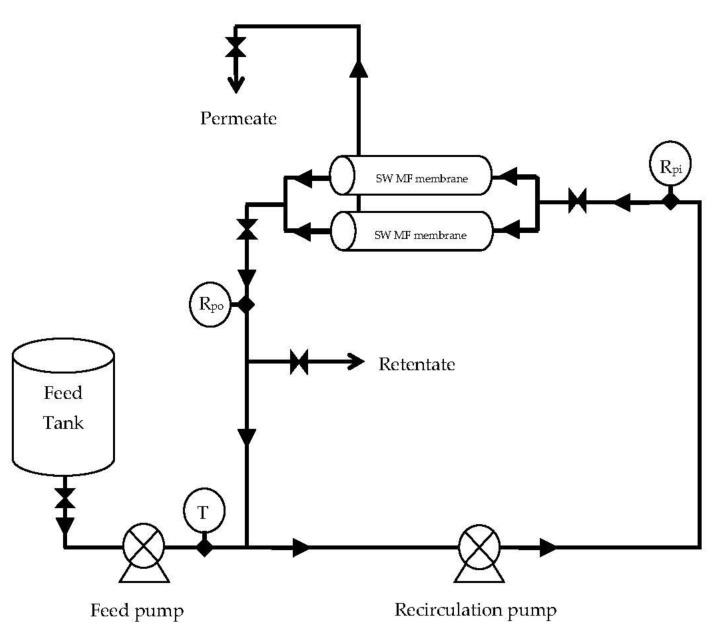
The spiral-wound microfiltration (SW-MF) system used during the microfiltration of skim milk at different pressures and different diafiltration (DF) levels. T = temperature; Rpi = retentate pressure inlet; Rpo = retentate pressure outlet.

Pilot-scale experiments were conducted with 151.4 L of fresh skim milk for each experiment (Figure 6). A batch type pilot MF unit fitted with two SW (3830) PVDF membrane elements was used for the MF of skim milk. Five pilot experiments were conducted at a 34.5 kPa baseline and 103.4 kPa differential pressures, resulting in a TMP of 86.2 kPa (12.5 psi). These five runs used 100% DF. Two experiments were conducted at the same pressures, but with 150% DF. Experiments were also conducted at a 62.1 kPa baseline and 103.4 kPa differential pressures, using 70 and 100% DF. All the experiments were conducted in continuous concentration mode, concentrating the feed to a final volume of 37.85 L giving a VR of 4. All the experiments were conducted at 24 °C. Deionized water was used for DF purposes.

Using the model developed for lab-scale experiments, SP removal for pilot runs operated under the above-mentioned operating conditions was predicted. Actual SP removal data obtained during the experiments were compared with the predicted values. From the data presented in Table 2, the model predicted SP removal within 90–95% of the actual values.

### 3.5. Pilot Plant Process Flux

**Figure 7:**
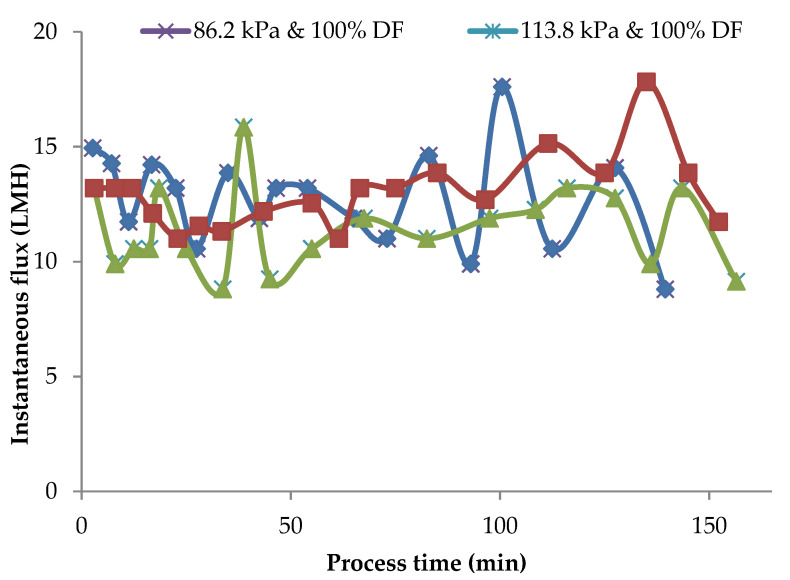
Instantaneous flux obtained for 86.2 kPa TMP at 100 and 150% DF and for 113.8 kPa and 100% DF. The data are from the pilot plant run.

In the MF of milk, it is essential to maintain the flux rates reasonably close to the initial flux rates for the selective fractionation of milk components. Any accumulation of a polarization layer on the membrane surface and consequent drop in flux rates is detrimental to the fractionation process. Instantaneous flux rates obtained during pilot-scale runs for 86.2 kPa TMP at 100 and 150% DF and for 100% DF at 86.2 kPa and 113.8 kPa at 100% DF are presented in Figure 7.

In these runs, DF water was added at VR of 1.23, 1.45, 1.68, 1.88, 1.88, and 2.67. After the final addition at a VR of 2.67, the process was continued to concentrate MCC to a VR of 4. The valleys and peaks in the flux graphs are due to the addition of DF water at different intervals. The controlled addition of DF water could maintain the flux at or close to initial flux for 80% of the total process run-time. In these pilot runs, the instantaneous flux was maintained within 90% of initial flux for about 80% of the process run. In the case of lab-scale experiments in which no DF was used, instantaneous flux dropped to about 60% of the initial value (Figure 2) with a consequent drop in flux and impairment of the selective removal of SP. The addition of DF water in small quantities over several intervals of the process run sustained the process flux rate for a longer time during the process and helped to maintain membrane selectivity.

### 3.6. Rejection of SP and CN-Pilot Runs

The data on CN/TKN and CN/TP ratios and the rejection of CN and SP are presented in Table 3. From the data, 86.2 kPa TMP had a higher SP removal rate and CN/TKN and CN/TP ratios, and a lower rejection of SP. The MF process is a pressure-sensitive process. The results show a definite advantage to operating at lower TMPs. Results also show the effect of the level of DF on SP removal and other parameters, with higher DF levels showing a definite advantage.

## 4. Conclusions

From the above-presented results, the use of DF positively affected process flux rates and the SP removal rate. Of the various DF levels used in the study, 150% DF resulted in the highest impact on the MF of skim milk. Over the range of operating pressures used in the study, the use of lower pressures resulted in higher SP removal and process flux rates. This study shows that the use of lower pressures and controlled degrees of DF (the addition of DF water at several intervals) results in maximized SP removal and process flux rates.

## Figures and Tables

**Table 1:** Mean (*n* = 3) data on casein-to-total-protein ratio (CN/TKN), casein-to-true-protein ratio (CN/TP), rejection of casein (Rej CN) and of serum protein (Rej SP) obtained from the lab-scale experiments.

DF ^1^ (%)	0			70			100			150		
TMP ^2^ (kPa)	34.5	62.1	103.4	34.5	62.1	103.4	34.5	62.1	103.4	34.5	62.1	103.4
**CN/TKN**	0.89 ^g^	0.89 ^g^	0.87 ^h^	0.94 ^b^	0.93 ^c^	0.9 ^f^	0.94 ^b^	0.93 ^c,d,e^	0.92 ^e^	0.96 ^a^	0.95 ^b^	0.93 ^c,d,e^
**CN/TP**	0.90 ^f^	0.90 ^f^	0.89 ^g^	0.94 ^b,c^	0.94 ^b,c^	0.92 ^e^	0.95 ^b^	0.95 ^b^	0.93 ^c,d^	0.96 ^a^	0.95 ^b^	0.93 ^c,d^
**Rej CN**	1.00 ^a^	0.98 ^a,b,c^	0.99 ^a,b^	0.98 ^a,b,c^	0.99 ^a,b^	0.99 ^a,b^	1.00 ^a^	0.98 ^a,b,c^	0.97 ^b,c^	0.98 ^a,b,c^	0.96 ^c^	0.97 ^b,c^
**Rej SP**	0.33 ^c^	0.40 ^b^	0.53 ^a^	0.14 ^e,f^	0.17 ^e^	0.32 ^c,d^	0.13 ^e,f^	0.17 ^e^	0.27 ^c,d^	0.10 ^f^	0.16 ^e,f^	0.25 ^d^

^1^ DF = diafiltration; ^2^ TMP = transmembrane pressure. ^a–h^ Mean values within same row not sharing a superscript are significantly different (*p* < 0.05).

**Table 2:** Comparison of SP removal predicted by the model and actual SP removal data obtained from the experiments.

Run #	Operating Pressure, kPa			DF ^1^, %	Serum Protein Removal, %		
	Base	Differential	TMP ^2^		Actual	Predicted	Difference
1	34.5	103.4	86.2	100	66.54	65.56	−0.98
2	34.5	103.4	86.2	100	68.20	65.56	−2.64
3	34.5	103.4	86.2	100	65.46	65.56	0.10
4	34.5	103.4	86.2	100	63.40	65.56	2.16
5	34.5	103.4	86.2	100	63.15	65.56	2.41
6	34.5	103.4	86.2	150	67.86	74.56	6.70
7	34.5	103.4	86.2	150	68.84	74.56	5.72
8	62.1	103.4	113.8	70	53.80	51.89	−1.91
9	62.1	103.4	113.8	70	52.70	51.89	−0.81
10	62.1	103.4	113.8	100	61.33	57.29	−4.04

^1^ DF = diafiltration; ^2^ TMP = transmembrane pressure.

**Table 3:** Mean ± SD of serum protein removal (SP R, %), casein-to-total-protein (CN/TKN), casein-to-true-protein (CN/TP) ratio, and rejection of casein (Rej CN) and serum protein (Rej SP) data obtained from pilot plant experiments.

Pressure	34.5 Base/86.5 kPa TMP ^2^		62.1 Base/113.8 kPa TMP ^2^	
DF ^1^, %	100	150	70	100
Item	Mean ± SD (*n* = 5)	Mean ± SD (*n* = 3)	Mean ± SD (*n* = 3)	-
SP R, %	65.4 ± 2.11	68.4 ± 0.64	53.3 ± 0.78	61.3
CN/TNK	0.92 ± 0.01	0.92 ± 0.00	0.90 ± 0.01	0.91
CN/TP	0.92 ± 0.01	0.93 ± 0.01	0.90 ± 0.00	0.92
Rej CN	0.98 ± 0.00	0.99 ± 0.01	0.99 ± 0.00	0.98
Rej SP	0.27 ± 0.02	0.27 ± 0.01	0.38 ± 0.01	0.30

^1^ DF = diafiltration; ^2^ TMP = transmembrane pressure.

## Data Availability

Data sharing not applicable.
